# Transcriptional profiling of embryos lacking the lipoprotein receptor SR-B1 reveals a regulatory circuit governing a neurodevelopmental or metabolic decision during neural tube closure

**DOI:** 10.1186/s12864-018-5110-2

**Published:** 2018-10-05

**Authors:** Nicolás Santander, Carlos Lizama, Leandro Murgas, Sebastián Contreras, Alberto J. M. Martin, Paz Molina, Alonso Quiroz, Katherine Rivera, Francisca Salas-Pérez, Alejandro Godoy, Attilio Rigotti, Dolores Busso

**Affiliations:** 1 Department of Nutrition, Diabetes, and Metabolism, School of Medicine, Pontificia Universidad Católica de Chile, Marcoleta 367, 83300024 Santiago, CP Chile; 2 Cardiovascular Research Institute, University of California, San Francisco, CA USA; 3 Network Biology Laboratory, Center for Genomics and Bioinformatics, Faculty of Sciences, Universidad Mayor, Santiago, Chile; 4 Faculty of Biological Sciences, Pontificia Universidad Católica de Chile, Santiago, Chile; 5 Department of Urology, Roswell Park Comprehensive Cancer Center, Buffalo, NY USA; 6 Center of Molecular Nutrition and Chronic Diseases, School of Medicine, Pontificia Universidad Católica de Chile, Santiago, Chile

**Keywords:** SR-B1, Neural tube defects, RNA-Seq, Androgen receptor, Gene expression

## Abstract

**Background:**

The high-density lipoprotein receptor SR-B1 mediates cellular uptake of several lipid species, including cholesterol and vitamin E. During early mouse development, SR-B1 is located in the maternal-fetal interface, where it facilitates vitamin E transport towards the embryo. Consequently, mouse embryos lacking SR-B1 are vitamin E-deficient, and around half of them fail to close the neural tube and show cephalic neural tube defects (NTD). Here, we used transcriptomic profiling to identify the molecular determinants of this phenotypic difference between SR-B1 deficient embryos with normal morphology or with NTD.

**Results:**

We used RNA-Seq to compare the transcriptomic profile of three groups of embryos retrieved from SR-B1 heterozygous intercrosses: wild-type E9.5 embryos (WT), embryos lacking SR-B1 that are morphologically normal, without NTD (KO-N) and SR-B1 deficient embryos with this defect (KO-NTD). We identified over 1000 differentially expressed genes: down-regulated genes in KO-NTD embryos were enriched for functions associated to neural development, while up-regulated genes in KO-NTD embryos were enriched for functions related to lipid metabolism. Feeding pregnant dams a vitamin E-enriched diet, which prevents NTD in SR-B1 KO embryos, resulted in mRNA levels for those differentially expressed genes that were more similar to KO-N than to KO-NTD embryos. We used gene regulatory network analysis to identify putative transcriptional regulators driving the different embryonic expression profiles, and identified a regulatory circuit controlled by the androgen receptor that may contribute to this dichotomous expression profile in SR-B1 embryos. Supporting this possibility, the expression level of the androgen receptor correlated strongly with the expression of several genes involved in neural development and lipid metabolism.

**Conclusions:**

Our analysis shows that normal and defective embryos lacking SR-B1 have divergent expression profiles, explained by a defined set of transcription factors that may explain their divergent phenotype. We propose that distinct expression profiles may be relevant during early development to support embryonic nutrition and neural tube closure.

**Electronic supplementary material:**

The online version of this article (10.1186/s12864-018-5110-2) contains supplementary material, which is available to authorized users.

## Background

The Scavenger Receptor class B type 1 (SR-B1) is a multiligand receptor that binds several classes of lipoproteins. It acts as the major receptor for high density lipoproteins (HDL) in the adult mouse [1]. SR-B1 binds HDL with high affinity and mediates selective, non-endocytic uptake as well as efflux of lipids by cells [2]. This receptor plays a key role in regulating circulating cholesterol levels because it is responsible for the clearance of plasma cholesterol in the liver for its excretion in bile [3]. In steroidogenic cells, such as adrenocortical cells and ovarian granulosa cells, SR-BI mediates the uptake of HDL-cholesteryl esters to be used a substrate for steroid hormone synthesis [4, 5]. Additionally, SR-B1 mediates the transport of other classes of lipids, including the lipophilic vitamins A, D, and E [6–9].

Besides its role in cholesterol homeostasis in adult animals, SR-B1 is involved in early development. Nearly 20 years ago, during the generation of SR-B1 knock out (KO) mice via heterozygous intercrosses, the proportion of weaned homozygous null mice was half that expected by the Mendelian ratio [10]. More recently, we identified the cause of this alteration to be neonatal lethality resultant of neural tube defects (NTD). Between 35 and 50% of SR-B1 KO mouse embryos analyzed in different cohorts failed to close their neural tube in the cranial region, and exhibited exencephaly, a congenital malformation that leads to perinatal death [11, 12]. Despite the striking embryonic phenotype observed in SR-B1 KO embryos, SR-B1 protein is not present in the embryo itself during neural tube closure. Instead, SR-B1 is localized in trophoblast giant cells [11, 13] surrounding the entire conceptus, that mediate the first step in maternal-fetal nutrient transport. Consistent with SR-B1 localization in cells mediating nutrient uptake, SR-B1 KO embryos show severe vitamin E deficiency [12]. Interestingly, maternal dietary supplementation with vitamin E completely rescues the NTD phenotype in KO embryos [12], underscoring the relevance of this vitamin in neural tube closure in this model.

Despite the biochemical insights described above, there is no information regarding the molecular determinants of NTD in SR-B1 KO embryos. In this work, we sought to identify molecular pathways that contribute to the phenotypic differences in normal vs. NTD SR-B1 KO embryos, and to analyze the effect of vitamin E on those pathways. We used transcriptional profiling by RNA-Seq in wild-type (WT), SR-B1 KO morphologically normal embryos (KO-N) and SR-B1 KO embryos with NTD (KO-NTD) to isolate specific genes and assign potential biological processes that may be altered in KO-NTD embryos. We also studied whether maternal vitamin E supplementation may prevent NTD by protecting embryos from aberrant gene expression. Using gene regulatory networks centered on the differentially expressed genes in KO-N and KO-NTD embryos, we uncovered a new regulatory circuit that may modulate neural tube closure in SR-B1 KO embryos. Our studies revealed a molecular basis to understand NTD generation in SR-B1 KO embryos, and provide new important insights that contribute to the understanding of NTD.

## Results

### Overall analyses of sequencing data in SR-B1 KO versus wild-type embryos

To investigate the potential molecular mechanisms underlying impaired neural tube closure in SR-B1 KO embryos, we performed an unbiased gene expression analysis by massive mRNA sequencing in E9.5 WT embryos and SR-B1 KO embryo with the two distinct phenotypes: KO-N and KO-NTD, retrieved from SR-B1 heterozygous intercrosses. To minimize variability among samples, we used pools of 3 female embryos for RNA extraction. We decided using only female embryos to avoid detecting the differential expression of genes in sex chromosomes (and potentially their downstream genes), that may result from using pools of male and female embryos. In each sample, we obtained over 40 million reads, representing over 2 Gbases sequenced with a mean quality score of 39.7 (Additional file 1: Table S1).

**Fig. 1:**
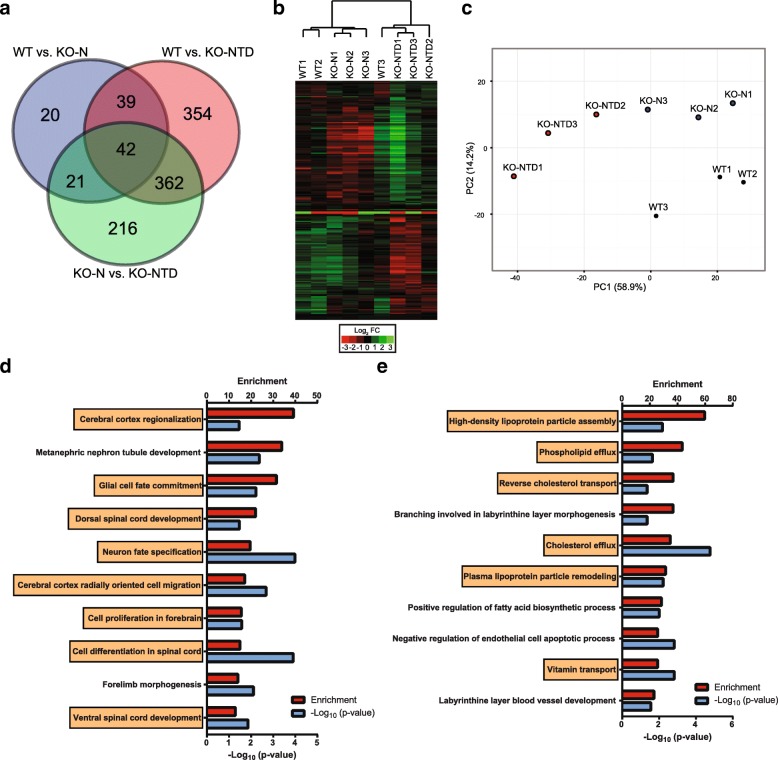
Divergent transcriptional profiles SR-B1 KO and wild-type embryos. **a**. Differentially expressed genes in each group of embryos are shown in a Venn diagram. **b**. Single genes with differential expression of over 2-fold were used to cluster samples hierarchically. The heatmap shows the fold change of the relative expression levels of each gene relative to the grand mean. **c**. Principal component analysis of each sample using all the differentially expressed genes. Percentage of the total variance explained by each principal component (PC) is shown in brackets. **d**. Gene set enrichment analysis of genes underexpressed in KO-NTD embryos compared to both WT and KO-N embryos. Processes related to neural development are shown in orange boxes. **e**. Enrichment analysis of genes overexpressed in KO-NTD embryos compared to WT and KO-N embryos. Processes involved in lipid metabolism are highlighted in orange boxes

Using the sequencing data, we performed differential gene expression analysis by the following pairwise comparisons: 1) WT vs. KO-N, 2) WT vs. KO-NTD and 3) KO-N vs. KO-NTD. We identified over 1,000 genes showing differential expression in at least one of the comparisons (Fig. 1a and Additional file 2). The number of differentially expressed genes in WT vs. KO-NTD and KO-N vs. KO-NTD was higher than in WT vs. KO-N. This suggests that, among the three groups, KO-NTD embryos are the most different. To test this objectively, we analyzed the data using hierarchical clustering and principal component analysis (Fig. 1b-c). These analyses show that the samples within each group tend to cluster together, and that WT and KO-N groups are closer from each other than to the KO-NTD group. Despite their similarity, WT and KO-N samples cluster separately and have 129 differentially expressed genes. These initial analyses showed that most of the differentially expressed genes are associated to the neural phenotype, since KO-NTD embryos have the most differentially expressed genes compared to WT and KO-N embryos.

### Functional categories of differentially expressed genes in SR-B1 KO versus wild-type embryos

To identify possible biological processes impaired in KO-NTD embryos that may contribute to their phenotype, we used enrichment analyses to associate differentially downregulated or upregulated genes in KO-NTD with functional categories likely to be impaired, or enhanced, respectively.

The list of genes that were downregulated in KO-NTD compared to WT and KO-N was enriched in genes with neurodevelopmental functions (Fig. 1d and Additional file 1 : Table S2): eight out of the ten top enriched Gene Ontology terms were related to neural development. Thus, a reduced expression in this subset of genes may contribute to the etiology of NTD in this mouse model.

The genes overexpressed in KO-NTD embryos were enriched in functions involved in nutrient transport and metabolism, as well as blood vessel development (Fig. 1e and Additional file 1: Table S3). Among the ten most enriched Gene Ontology terms in this gene set, six corresponded to lipid and vitamin transport processes. This transcriptional signature may represent an attempt from SR-B1 KO embryos to increase the uptake of vitamins and other lipids in order to compensate for inefficient lipid transport due to SR-B1 deficiency, as observed for vitamin E [12].

### Identification of candidate genes responsible for the different phenotypes in KO embryos

Using the information obtained from the enrichment analyses on functional categories altered in KO-NTD embryos, we prepared complete lists of all the genes involved in specific biological processes of our interest, and compared them to the lists of down- or upregulated genes in KO-NTD vs KO-N and WT embryos. The aim of this strategy was to identify pathways that may be key determinants of neural tube closure success in SR-B1 KO embryos.

**Fig. 2:**
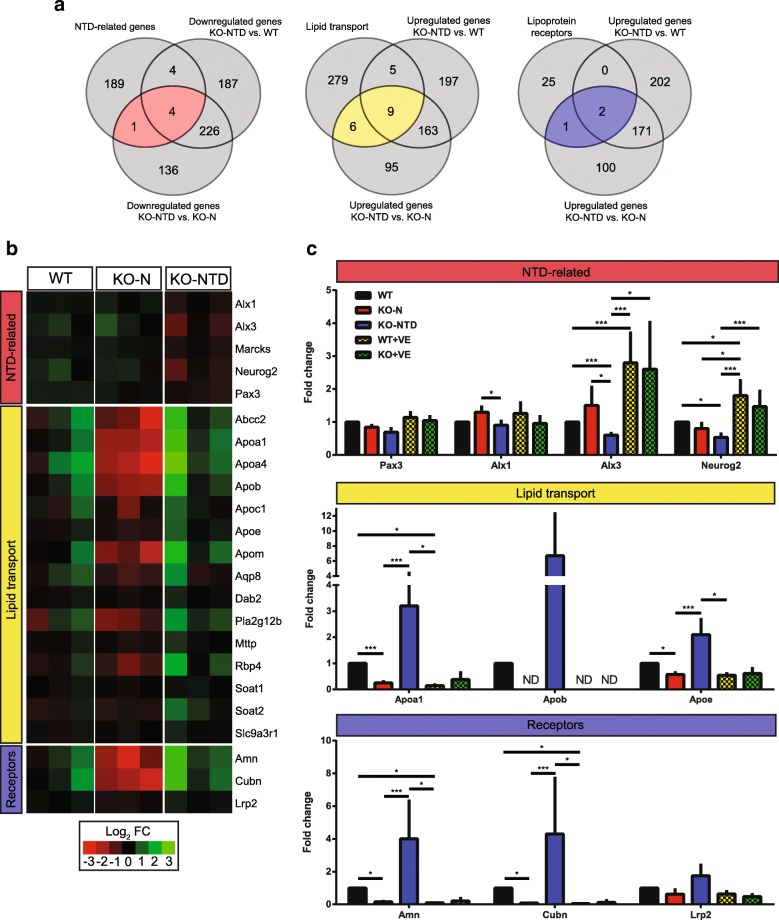
Identification of candidate genes infuencing the phenotype of KO-NTD embryos. **a**. Genes involved in specific biological processes with differential expression are shown in Venn diagrams. **b**. Heatmap showing relative expression levels of the genes highlighted in the center of the Venn diagrams, based on the RNA-Seq data. The scale shows logarithmic fold change from the grand mean. **c**. rtPCR analysis of mRNA levels of the genes shown in (**a**) in embryos from dams fed control chow or a vitamin E-supplemented diet. *N* = 3 per group. ND: not detected. *p < 0.05, ***p < 0.001; Pair-Wise Fixed Reallocation Randomisation Test

First, among genes that were downregulated in KO-NTD embryos, we sought to analyze those previously associated to neural tube closure. We used a list of genes whose inactivation has been associated with NTD in mice (https://figshare.com/articles/Genes_NTD_wiki_txt/7139354) that is maintained and curated by Dr. Lee Niswander’s group (see Methods for further information). Four genes downregulated in KO-NTD embryos -compared to WT and KO-N embryos- were found in the list of genes associated with NTD (Fig. 2a, b): ALX homeobox 3 (*Alx3*), myristoylated alanine-rich C kinase substrate (*Marcks*), neurogenin 2 (*Neurog2*), and paired box 3 (*Pax3*). Furthermore, ALX homeobox 1 (*Alx1*) was downregulated in KO-NTD embryos compared to KO-N embryos. With the exception of *Marcks*, all these genes encode for transcription factors expressed in the neural tube or the adjacent mesenchyme. In the sequencing data, we observed different patterns of expression of these genes in WT and KO-N embryos. *Alx3* and *Alx1* appear to be overexpressed in KO-N embryos compared to WT embryos, whereas *Neurog2* and *Pax3* seemed to be downregulated (Fig. 2b).

We validated the results from the sequencing data analyses for transcription factors using real-time PCR (rtPCR) to quantify mRNA levels for those genes in independent biological samples. With some exceptions, the overall pattern of expression obtained by rtPCR was similar to that observed by massive RNA sequencing. The differences in mRNA levels were not statistically significant in all cases. We validated the differentially reduced expression of *Alx1* and *Alx3*, while the levels of mRNA of *Pax3* were not statistically different between the groups (Fig. 2c). Finally, levels of *Neurog2* mRNA in KO-NTD embryos were statistically lower than those in WT embryos, but not from those in KO-N embryos (Fig. 2c).

We also tested by rtPCR the expression of the 4 NTD associated genes in embryos retrieved from dams fed a α-tocopherol-enriched diet that prevents NTD in SR-B1 KO embryos [12]. Embryos from vitamin E supplemented females showed higher mRNA levels for Alx3 and Neurog2 in WT (WT + VE) and morphologically normal SR-B1 KO (KO + VE) embryos, reaching levels over control values (Fig. 2c) and suggesting that changes in the expression of neural tube-related genes may contribute to the preventive effect of α-tocopherol on NTD.

Besides analyzing genes involved in NTD, we constructed a second list of genes including those involved in lipid transport or mobilization and genes encoding for lipoprotein receptors and compared them to the lists of upregulated genes in KO-NTD vs KO-N and WT embryos. As mentioned previously, we hypothesized that upregulation of genes in that functional categories may represent compensatory mechanisms aimed at increasing the flux of lipids towards the embryo in the absence of SR-B1. Results showed that apolipoproteins involved in HDL metabolism (Fig. 2a, b), including structural apolipoproteins (ApoA-I and ApoE) and proteins conferring non-canonical functions to HDL, such as inhibition of inflammation, oxidative stress and retinol transport (ApoA-IV, ApoM, and RBP) were upregulated in KO-NTD embryos. We also observed upregulation of the *Apob* gene, which encodes for the major apolipoprotein in larger (non-HDL) lipoproteins, and of the *Apoc1* gene, encoding one of the apolipoproteins normally contained within triglyceride-rich lipoproteins. Levels of mRNA coding for proteins involved in lipid packaging into lipoproteins within the endoplasmic reticulum (MTTP, SOAT1, SOAT2) were also higher in KO-NTD embryos. With regard to differential expression of lipoprotein receptors, genes encoding for members of the multiligand complex formed by AMN, CUBN, and LRP-2 were upregulated (Fig. 2a, b). Interestingly, mRNA levels for the subset of genes involved in lipid transport were upregulated in KO-NTD embryos yet showed very low mRNA levels in KO-N embryos compared to the WT group (Fig. 2b).

We selected structural apolipoprotein genes (*Apoa1*, *Apob*, and *Apoe*) and members of the multiligand endocytic complex (*Amn*, *Cubn*, and *Lrp2*) for further analysis using rtPCR. We validated the strongly divergent mRNA levels of *Apoa1*, *Apob*, *Apoe*, *Amn*, and *Cubn* in KO-N versus KO-NTD embryos (Fig. 2c). *Lrp2* mRNA levels showed the same trend to that observed in the RNA-Seq data, but the differences did not reach statistical significance. Importantly, maternal treatment with α-tocopherol in WT and SR-B1 KO embryos was associated with mRNA levels similar to those observed in control KO-N embryos for all the genes analyzed (Fig. 2c).

In summary, these studies showed strongly divergent expression of specific genes in KO-N and KO-NTD embryos that may explain the partially penetrant neural phenotype in these otherwise genetically identical embryos. Interestingly, normalization of the mRNA levels of these genes was observed in embryos retrieved from dams supplemented with α-tocopherol, providing a plausible mechanism by which this treatment prevents NTD in SR-B1 KO embryos.

### Effect of defective HDL-associated apolipoprotein expression on the proportion of SR-B1 KO mice at weaning

Since we observed higher mRNA levels for genes encoding for HDL apolipoproteins in KO-NTD than in KO-N embryos, we next evaluated the effect of genetic reduction of HDL-associated apolipoproteins on NTD in SR-B1 KO embryos. We hypothesized that a lower incidence of NTD would occur in apolipoprotein-deficient SR-B1 embryos, and used genotype data from two mouse colonies available in our animal facility that are both SR-B1 and ApoA-I or ApoE deficient. Since NTD embryos die soon after birth, the proportion of SR-B1 KO mice is reduced by nearly 50% at weaning [11, 10]. Therefore, changes in the incidence of the NTD phenotype may be reflected in the proportion of SR-B1 KO mice recovered at weaning. We analyzed the proportion of SR-B1 KO mice weaned from two types of intercrosses: 1) SR-B1 KO males and SR-B1 heterozygous females, both *Apoa1* deficient [ApoA-I KO/SR-B1 KO x ApoA-I KO/SR-B1 heterozygous] and 2) male and female SR-B1 heterozygous mice expressing very low levels of ApoE [ApoeR61^h/h^/SR-B1 het x ApoeR61^h/h^/SR-B1 het) [14]. We decided to use SR-B1 KO males in the former intercross, because this double transgenic line breeds poorly and this strategy allowed us to increase the number of SR-B1 KO offspring for our analyses. As controls, we used crosses of SR-B1 KO males with SR-B1 heterozygous females or intercrosses of SR-B1 heterozygous mice. We assumed that any change in the proportion of weaned mice lacking SR-B1 in the double mutants should stem from changes in the incidence of NTD, but we did not evaluate the embryos directly.

**Fig. 3:**
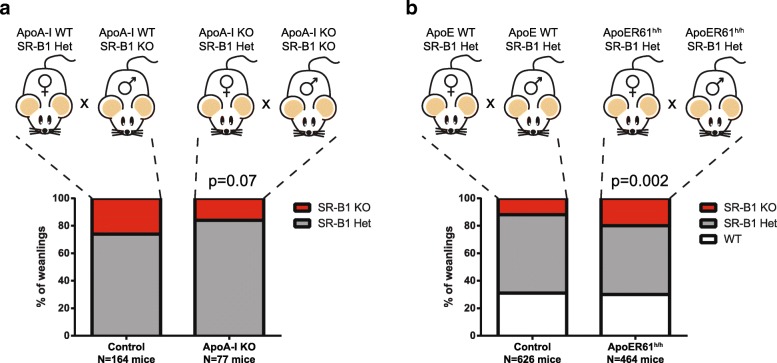
Effect of genetic manipulation of apolipoprotein genes on SR-B1 genotype proportion at weaning. We determined the effect of inactivating ApoA-I (**a**) or reducing ApoE levels (**b**) on the proportion of SR-B1 KO pups recovered at weaning. The breeding schemes are shown above and the percentages obtained for each possible SR-B1 genotype are shown below. The *p*-values were calculated using Fisher’s exact test (**a**) or χ^2^ test (**b**)

We analyzed genotype proportions in the offspring of ApoA-I KO/SR-B1 KO x ApoA-I KO/SR-B1 heterozygous pairs and control SR-B1 KO x SR-B1 heterozygous pairs. As expected, the proportion of SR-B1 KO mice in a normal ApoA-I background weaned was around 25%, which is half of the expected from the Mendelian ratio of 1:1. In the litters obtained from ApoA-I deficient mice, we observed a reduction in the proportion of SR-B1 KO pups at weaning from 26 to 15% (Fig. 3a). Although this difference was not statistically significant (*p* = 0.07; Fisher’s exact test), the reduced yield of ApoA-I/ KO/SR-B1 KO mice in the ApoA-I deficient colony suggests that inactivation of the Apoa1 gene may increase the susceptibility to NTD in embryos. These results suggest that ApoA-I may be protective against NTD in SR-B1 KO embryos.

To extend this observation to embryos expressing normal SR-B1 levels, we evaluated whether ApoA-I KO (without SR-B1 deficiency) embryos show NTD. Among 8 litters, we observed the presence of exencephaly - the developmental consequence of cephalic NTD – in 2 out of 48 fetuses in E18.5, indicating that ApoA-I deficiency is also associated with a low incidence of NTD in embryos expressing normal levels of SR-B1.

We next compared the proportions of ApoeR61^h/h^/SR-B1 heterozygous intercrosses and control SR-B1 heterozygous intercrosses. As expected, the proportion of SR-B1 KO in the control pairs was half of that expected by the Mendelian proportion. In ApoeR61^h/h^/SR-B1 heterozygous intercrosses, the reduction of ApoE levels was associated with an increased proportion of SR-B1 KO pups at weaning from 13 to 20% (*p* = 0.002; χ^2^ test) (Fig. 3b). These results support our hypothesis and suggest that reduced ApoE expression may partially prevent NTD in SR-B1 KO embryos.

### Regulators of the transcriptional profiles in SR-B1 KO embryos

Our transcriptomic data showed divergent transcriptional profiles in KO-N and KO-NTD embryos, despite identical genotypes. To identify possible regulatory factors driving these differences, we constructed and analyzed gene regulatory networks based on the differentially expressed genes in KO-N vs. KO-NTD embryos. We obtained the background mouse regulatory network from the RegNetwork database [15], using data based on experimental evidence. Next, we constructed a regulatory network using genes with differential expression in KO-N vs. KO-NTD embryos plus their transcriptional regulators (Additional file 3: Script 1 and Additional file 4). We systematically filtered this general network (see Methods and Additional file 5) using those genes we previously analyzed by rtPCR (genes of interest) to obtain subnetworks related to the specific biological processes altered in KO-NTD embryos. Since the general network was constructed with data based on experimental evidence, it contained only a subset of the genes of interest and subnetworks were constructed using *Apoa1*, *Apob*, *Apoe*, and *Pax3* as the quadruplet of seed nodes. The basic topological properties of these subnetworks, our general network (KO-N vs. KO-NTD network), and the background network from RegNetwork are shown in Additional file 1: Table S4.

**Fig. 4:**
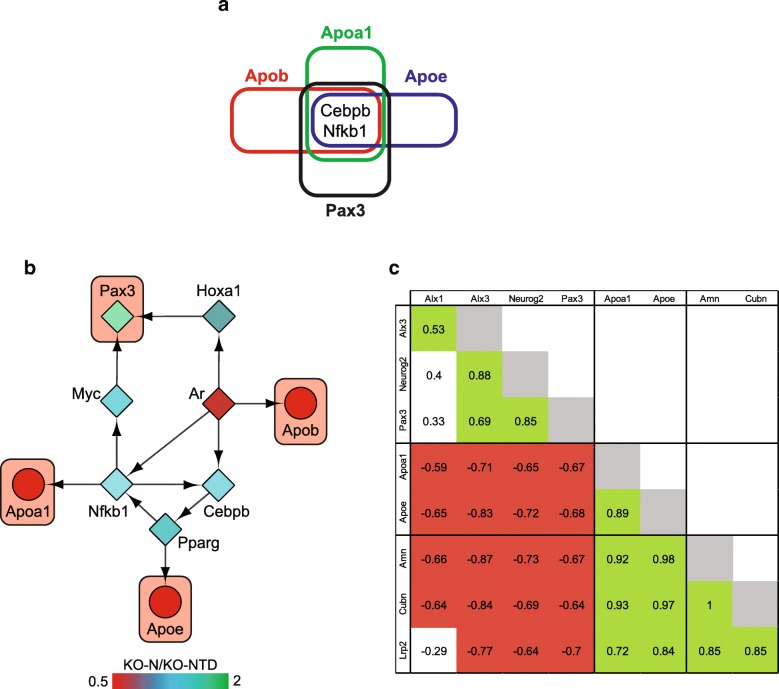
Shared transcriptional regulation of NTD-related and lipid metabolism genes. **a**. Shared transcription factors in the gene regulatory subnetworks of *Apoa1*, *Apob*, *Apoe*, and *Pax3*. **b**. Regulatory relationships connecting members of the regulatory circuit and the genes of interest through the shortest paths. **c**. Pearson correlation coefficients of mRNA levels of the genes of interest within each individual embryo assayed (*N* = 15). Colored cells indicate statistically significant correlations (*p* < 0.05). Green cells show positive correlation, while red cells have negative correlations

The transcription factors shared in all the subnetworks were defined as possible regulators driving differences in transcriptomic profiles of KO-N versus KO-NTD embryos. There were two transcription factors present in all four subnetworks (Fig. 4a): CCAAT/enhancer binding protein beta (*Cebpb*) and nuclear factor of kappa light polypeptide gene enhancer in B cells 1, p105 (*Nfkb1*). To formally test for the probability of observing as many shared transcription factors between any four subnetworks constructed using our strategy, we used the same protocol to build all the possible quadruplets of subnetworks within our general network (Additional file 3: Script 2 and Script 3). We used any combination of seed nodes or only one transcription factor and 3 non transcription factors, to model the specific combination of seed nodes among the genes of interest. This approach allows us to examine the true distribution of shared transcription factors among quadruplets within our network and to derive the exact probability of the occurrence of any result. Whether we started with any combination of seed nodes or with only one transcription factor, only 27% of the quadruplets shared as many transcription factors as the genes of interest (Additional file 1: Figure S1a-b), indicating that this is an uncommon finding in the whole network.

To analyze if the transcriptional differences observed between KO-N and KO-NTD embryos may potentially be explained by the shared transcription factors mentioned above, we traced the shortest regulatory paths from the genes of interest back to *Cebpb* or *Nfkb1* within the general network, without considering the directionality of the regulatory relationships. This analysis showed that the genes of interest were regulated, directly or indirectly, by at least one of the components of the regulatory circuit (Fig. 4b). Interestingly, three transcription factors were sufficient to explain the regulation of the genes of interest: the androgen receptor (*Ar*), *Cebpb*, and *Nfkb1*. Indirect regulations required only one intermediate node. We then analyzed if the distances of these transcription factors to the genes of interest were relatively short, compared to all the transcription factors within the general network. We traced the shortest paths starting from all the transcription factors to each of the genes of interest and calculated the sum of these distances (Additional file 3: Script 4). Only ten transcription factors had a directional path towards all the genes of interest in the general network. *Ar* and peroxisome proliferator-activated receptor alpha (*Ppara*) showed the lowest sum of the shortest paths with a total distance of 8, while *Nfkb1* summed a total distance of 9 (Additional file 1: Table S5). These data show that the regulatory circuit formed by *Ar*, *Cebpb*, and *Nfkb1* might be important in shaping the transcriptional profile observed in SR-B1 KO embryos. Importantly, *Ar* appears to be the master regulator of these interactions, while *Cebpb* and *Nfkb1* seem to act as integrators of the information. Also, *Ar* is the only regulator showing different mRNA levels in KO-NTD embryos compared to KO-N embryos in the RNA-Seq data, although this difference did not reach adjusted statistical significance (Additional file 1: Figure S2).

Since the genes of interest appear to share transcriptional regulators in our model system, we predicted that they should show correlations in their mRNA levels, independently of the genotype or phenotype. In order to test this idea in all the scenarios posed by our model, we used the rtPCR data for all the genes of interest in embryos obtained from control and vitamin E-supplemented dams. We did not include data of *Apob* in this analysis, because its encoding mRNA was not detected in three of the study groups and, therefore, the correlation could not be evaluated. We assessed the correlation between the mRNA levels of each gene of interest and all the other genes of interest within each embryo. Using this strategy, we detected correlations of varying strengths in the mRNA levels of all the genes of interest (Fig. 4c). Interestingly, genes from the same categories (i.e., NTD- and lipoprotein metabolism-related) showed positive correlations, whereas genes from different categories showed negative correlations. Additionally, we used the online tool at http://marionilab.cruk.cam.ac.uk/organogenesis/ [16] (accessed on 02-05-2018) to define the approximate expression domains of the genes of interest and the members of the regulatory circuit. Almost all the genes of interest and the members of the regulatory circuit are expressed in the neural tube or in the adjacent mesenchyme at the developmental stage prior to neural tube closure (Additional file 1: Figure S3). These results suggest that the genes of interest are co-regulated, supporting the hypothesis that the regulatory circuit identified herein may control their mRNA levels.

### Role of the androgen receptor in the transcriptional profile of SR-B1 KO embryos

**Fig. 5:**
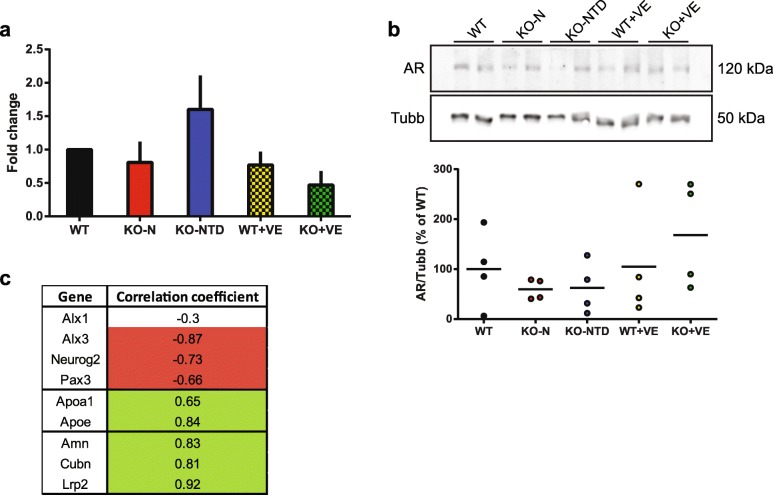
Characterization of androgen receptor expression. **a**. mRNA levels of the androgen receptor were analyzed by rtPCR in embryos with different genotypes from control or vitamin E-supplemented dams (*N* = 3 per group). **b**. Representative blot showing the abundance of the androgen receptor in whole embryo lysates. Quantification is shown below (*N* = 4 per group in 2 independent blots). **c**. Correlation between mRNA levels of the androgen receptor and the genes of interest within each embryo (*N* = 15). Colored cells indicate statistically significant correlations (*p* < 0.05). Green cells show positive correlation, while red cells have negative correlations

Given the central role of AR in the regulatory circuit identified in this work, and considering that this receptor was previously localized to the neuroepithelium, along the neural tube, in E9.5 mice [17], we next studied its mRNA and protein levels in embryos from the different groups, using rtPCR and western blotting, respectively. At the mRNA level, we observed an increase in KO-NTD embryos compared to the embryos from all the other groups, but these differences did not reach statistical significance (Fig. 5a). Similarly, we did not observe statistically significant differences in the abundance of the AR protein in whole embryo lysates (Fig. 5b). Future studies designed to evaluate the expression domain and the transcriptional activity of Ar in SR-B1 KO embryos prior to neural tube closure will provide information regarding the contribution of an abnormal Ar activity to NTD.

As a preliminary approach, we took advantage of the rtPCR data and assessed the correlation between the mRNA levels of Ar and all the genes of interest in each embryo of all the study groups. We used rtPCR instead of the Western blot data, because mRNA levels for all the genes were measured in the same set of embryos. By contrast, AR protein abundance was evaluated in an independent set of samples. We observed intermediate to strong correlations in the mRNA levels of *Ar* with each of the genes of interest, with the exception of *Alx1*, which showed no correlation (Fig. 5c). Interestingly, the direction of the correlations were opposite in the different gene categories, showing an inverse association for NTD-related genes and a positive relationship for lipoprotein metabolism genes. These findings support AR as an important transcriptional regulator of the genes of interest that may be contributing to the distinct transcriptional profiles observed in SR-B1 KO embryos.

### Presence of the NTD regulatory circuit in humans

Since we identified a new regulatory circuit potentially modulating neural tube closure in SR-B1 KO mouse embryos, we next studied if this circuit may regulate the expression of orthologs in humans. We obtained the nodes included in the murine regulatory circuit and the nodes of the genes of interest from the human background network in RegNetwork and assessed their regulatory relationships. The human background network based on experimental data included the nodes of *AR*, *NFKB1*, *APOA1*, *APOB*, and *APOE*, but not of any NTD-related gene. We observed a regulatory circuit involving *AR* and *NFKB1* that directly regulates one or more apolipoprotein genes (Additional file 1: Figure S4). In addition, *AR* directly regulates the expression of all the apolipoprotein genes contained in the network. This indicates that these genes indeed can define a regulatory circuit in humans and that it may serve a similar role as in mice.

## Discussion

Neural tube closure is a complex process involving the temporal and spatial regulation of hundreds of genes that support the highly regulated and dynamic events occurring concomitantly in the embryo [18, 19]. In turn, gene expression may adapt to changes in the maternal environment. The complexity of neural tube formation is illustrated by the large number of genes whose inactivation is associated with NTD [18] and poses a challenge to study this key event during embryo development.

Transcriptional profiling by microarray has been an important tool in elucidating the molecular pathways controlling this process for over a decade, and massively parallel sequencing has been used more recently in this regard [20, 21]. In this work, we used transcriptomic profiling by RNA-Seq to gain insights on underlying molecular determinants of NTD in mouse embryos lacking the HDL receptor SR-B1. We observed strikingly divergent transcriptional profiles in KO-N and KO-NTD embryos, involving differential expression of two sets of genes, one involved in neural tube closure and the other related to lipoprotein metabolism. Furthermore, we uncovered a new gene regulatory circuit that might be responsible for this divergent expression, and identified the androgen receptor as a possible master regulator of these transcriptional profiles potentially linked to impairment of neural tube closure.

Several lines of evidence have shown that neural tube closure depends on an adequate supply of different types of nutrients. Folic acid is broadly used to prevent NTD in humans, and several mouse models of this malformation are responsive to folate [22]. Vitamin E has been also used as an effective strategy for prevention of NTD in different rodent models [12, 23], in addition to several other nutrient-based preventative strategies [24, 25]. In our transcriptomic dataset, we observed a marked increase in the expression of several genes involved in nutrient uptake in KO-NTD embryos, in particular in genes important for HDL metabolism and function. This transcriptional response may represent an attempt to increase flux of HDL associated lipid molecules to the embryo, since SR-B1 KO embryos are severely vitamin E-deficient [12]. A similar transcriptional response has been previously observed in a mouse model of genetic folate deficiency-based NTD, the Reduced Folate Carrier 1 (RFC1) KO mouse [26], suggesting that this may be an adaptive mechanism to cope with an inadequate nutrient supply. This compensatory transcriptional adaptation to nutrient deprivation is characterized by upregulation in genes encoding members of the multiligand endocytic complex, including AMN, CUBN, and LRP-2. These proteins can form a plasma membrane bound complex that recognizes different ligands and mediates their endocytosis, and appear to be important for embryonic nutrition since preimplantation development until placentation [27]. Accordingly, inactivation of both *Cubn* and *Lrp2* causes embryonic lethality [28, 29]. In the embryo, this multiligand complex mediates the uptake of HDL and folate [27, 30], supporting the idea that their overexpression in RFC1 KO and SR-B1 KO-NTD embryos may represent a compensatory response to nutrient deficiency.

The transcriptional profile of KO-NTD and RFC1 KO embryos is also characterized by altered expression of several genes encoding for proteins that are HDL structural components or are associated to this lipoprotein class. The former category includes proteins with structural and receptor-binding functions, such as ApoA-I and ApoE, whereas the latter involves non-canonical HDL bound proteins, unrelated to lipid metabolism, such as transferrin and transthyretin. In this work, we provide evidence that overexpression of at least some of these genes is associated with the NTD phenotype in SR-B1 KO embryos.

Our studies on the proportions of SR-B1 KO offspring weaned from ApoA-I or ApoE- deficient mice provided further information on the potential relevance of HDL components for neural tube closure, but it should be noted that we did not evaluate NTD directly so we cannot rule out that double-mutant embryos have additional defects leading to lethality. Although high mRNA levels for ApoA-I were detected in KO-NTD embryos, the lack of ApoA-I in SR-B1 KO embryos did not protect them from NTD. ApoA-I KO/SR-B1 KO mice were weaned at a lower proportion than SR-B1 KO mice. Among other possibilities to explain this unexpected result, ApoA-I may be required for SR-B1 independent HDL-mediated transport of lipids or other non-canonical functions (e.g. cellular signaling regulation, antioxidant or anti-inflammatory properties), as both dams and the offspring in the matings analyzed lack ApoA-I. By contrast, the yield of ApoeR61^h/h^/SR-B1 KO mice at weaning was high compared to the yield of SR-B1 KO mice, suggesting that low ApoE levels may be protective against NTD in SR-B1 KO embryos. The mechanisms explaining the potentially negative influence of ApoE on neural tube closure are unclear. One possibility is that lipoprotein-associated ApoE modulates the lipid and/or protein content in those particles, negatively affecting their function(s). Alternatively, ApoE may exert a pro-apoptotic effect dependent on LRP-8 signaling, similar to that recently described in immune cells [31]. Since LRP-8 is detected in cells of the neural tube and the mesodermal lineage [16], ApoE may cause excessive apoptosis in the neural tube or the adjacent tissues of SR-B1 KO embryos, further impairing neural tube closure.

Our analyses of the gene regulatory networks associated with NTD in SR-B1 KO embryos led us to identify the androgen receptor as a possible new regulator of neural tube closure. Interestingly, the regulatory circuit governed by the androgen receptor appears to function similarly in mice and humans. The mRNA levels of this receptor were higher in SR-B1 KO-NTD embryos and these levels correlated strongly with the expression of the genes of interest. We propose that stochastic changes in *Ar* expression, as previously reported for several genes during embryo development [32, 33], may lead to differential susceptibility to NTD in embryos by modulating their transcriptional profile. In this scenario, high AR transcriptional activity may enhance a gene expression profile, favoring lipoprotein metabolism at the expense of reducing the expression of genes important for neural tube closure. This altered transcriptional profile would confer the embryo with increased susceptibility to NTD, which is only manifested phenotypically under presence of a “second hit”. In the case of SR-B1 KO embryos, this “second hit” may be vitamin E deficiency. Indeed, some vitamin E metabolites can reduce AR-dependent signaling [34, 35] suggesting that adequate vitamin E provision in the embryo may attenuate AR upregulation during neural tube closure. Although neural tube closure was not reported in transgenic mice overexpressing the AR, this phenotype was not directly evaluated and may have been missed during its generation or breeding [36]. Future studies will allow to explore the impact of concomitant AR upregulation and vitamin E deficiency in SR-B1 KO embryos on the incidence of NTD.

## Conclusions

In the present work, we identified possible molecular determinants of NTD in SR-B1 KO embryos and uncovered a gene regulatory circuit that may be involved in the differential regulation of the transcriptional response that modulates neural tube closure. From this circuit, the androgen receptor emerges as a possible new master regulator of a dichotomous transcriptional profile in early SR-B1 KO embryos during neural tube development. Future research will directly test the role of the androgen receptor in neural tube closure and the precise mechanisms involved as well as their potential extrapolation to humans.

## Methods

### Animals

SR-B1 KO mice, carrying the null mutation in the SR-B1 locus, were maintained on a mixed C57Bl6/J × 129 background (B6;129S2-Scarb1^tm1Kri^/J [1]). These mice as well as those carrying the hypomorphic Apoe allele (ApoeR61^h/h^) were provided by Dr. Monty Krieger from Massachusetts Institute of Technology in Cambridge, MA, USA [37]. ApoA-I KO/SR-B1 KO and apoA-I KO/SR-B1 het mice, carrying the null mutation in the Apoa1 gene (Apoa1^tm1Unc^), were obtained from crossing ApoA-I KO mice originally obtained from Jackson Laboratories (Bar Harbor, ME) with SR-B1 heterozygous mice. Animals were kept in plastic cages in the animal facility of the School of Medicine, Pontificia Universidad Católica de Chile at 25 °C and with a 12 h light:dark cycling, and consumed standard chow (Prolab RMH3000, Labdiet; 75 IU vitamin E/kg) and water ad libitum.

Pregnancies were generated by mating 2–4 month old SR-B1 heterozygous females with 2–6 month old SR-B1 heterozygous males. Female mice were checked daily for the presence of a copulatory plug during the first hour of the light cycle, the detection of which was marked as E0.5. Maternal dietary supplementation was done as previously reported [12]. All embryos were collected on day E9.5, when neural tube closure is complete in all wild-type embryos. Pregnant dams were anesthetized with a mixture of ketamine:xylazine (0.18 mg:0.012 mg per gram of body weight), blood was collected from the abdominal vena cava, the uteri were recovered, and mice were euthanized by cervical dislocation. Implantation sites were individually retrieved and embryos, parietal yolk sacs, and visceral yolk sacs were dissected. Neural tube closure was assessed in embryos and individual genotyping was performed using the visceral yolk sac as described [11].

Protocols were conducted in agreement with the National Research Council (NRC) publication Guide for Care and Use of Laboratory Animals (copyright 2011, National Academy of Science). All the studies were approved by the Ethics Committee for Animal Welfare from the School of Medicine of the Pontificia Universidad Católica de Chile (protocol #13–042).

### RNA extraction

Total RNA was obtained from three female embryos pooled together, or from individual embryos of unknown sex using the PureLink RNA Micro Kit (Invitrogen, CA), according to manufacturer’s instructions. Pooled embryos came from nine litters of dams fed control chow. DNA was eliminated by incubation with DNAse I (Sigma, MO) following instructions of the manufacturer. RNA integrity was evaluated in samples used for massively parallel sequencing, with the Bioanalyzer 2100 (Agilent, CA) and the Eukaryote Total RNA Nano assay (Agilent, CA). All the samples had a RNA Integrity Number of 10.

### mRNA sequencing

Strand-specific RNA-seq libraries were generated for each sample from 500 ng of total RNA using the Kapa Stranded mRNA-seq kit (Kapa Biosystems, South Africa) after poly-A enrichment according to the instructions of the manufacturer. The consistent fragment lengths of the sequencing libraries were ascertained using a Bioanalyzer 2100 micro-capillary gel electrophoresis instrument (Agilent, CA). The barcoded libraries were quantified by fluorometry on a Qubit instrument (Life Technologies, CA), and pooled in equimolar ratios. The pool was quantified by qPCR with a Kapa Library Quant kit (Kapa Biosystems, South Africa) and sequenced on one lane of an Illumina HiSeq 4000 sequencer (Illumina, CA) run with single-end 50 bp reads.

### Sequence analysis

Raw sequence quality was assessed using FastQC, and single end reads were groomed using FASTQ groomer 1.04. Reads were then mapped to the reference mouse genome version mm10 using TopHat2 0.7. The resulting alignment files were used to estimate the abundance of gene coding transcripts in FPKM, and to test the statistical significance of the differential expression with Cufflinks 2.2.1. All the analysis were done in the Galaxy platform [38] using a dedicated server.

### Hierarchical clustering and principal component analysis

Hierarchical clustering of individual samples was performed with Cluster 3.0 [39] using genes showing over 2-fold change. Data was log transformed and centered on the mean before clustering samples using Spearman Rank Correlation and Average linkage. Dendrogram and heatmaps were visualized using Java Treeview [40]. Principal component analysis was performed with the online tool ClustVis [41] with the same dataset.

### Functional annotation

Gene enrichment analysis was performed using the online tool PANTHER [42, 43], separating differentially expressed genes by down- or upregulation in the KO-NTD group.

Gene lists of specific functions were constructed as follows: genes involved in neural tube closure were obtained from the list maintained by Dr. Lee Niswander in https://figshare.com/articles/Genes_NTD_wiki_txt/7139354 (recovered on July, 2017). Genes involved in lipid transport were retrieved from Gene Ontology database, using the functional term “Lipid transport”. Lipoprotein receptors were identified as described [12]. A gene list constructed from Gene Ontology using the terms “Lipoprotein particle receptor activity” and “Regulation of plasma lipoprotein particle levels” was curated manually to include only those genes with experimental evidence of encoding lipoprotein binding receptors.

### Gene regulatory network analyses

A reference gene regulatory network was obtained from RegNetwork [15] retrieving only regulatory interactions with high quality experimental evidence. This regulatory network was filtered using the RNA-Seq data: interactions were only maintained if the gene coding for a transcription factor was detected in embryos, the genes showed differential expression, and the log_2_ of the fold change was 0.4 or more. This was done for each biological replicate using averaged expression from each technical replicate based on a custom script (Additional file 3: Script 1).

The general network was systematically filtered using criteria defined a priori. We used each gene of interest as a seed node and selected their neighbors, up to two levels. This subnetwork was isolated and filtered as follows: first, all the nodes with less than two connecting edges were eliminated; second, all the nodes not representing transcription factors were erased, with the exception of the seed node. After all the subnetworks corresponding to the genes of interest were created, we determined all the shared nodes. This process was repeated programmatically with all the possible quadruplets in the general network to reveal the distribution of shared transcription factors (Additional file 3: Script 2 and Script 3). These scripts select 4 nodes systematically, construct the corresponding subnetworks, and determine the number of shared transcription factors within them. Then, they produce the distribution of the number of shared transcription factors in the general network as output.

To identify the nearest putative regulators to the genes of interest in the general network, we determined the shortest paths from each transcription factor in this network to each of the genes of interest, considering the directionality of the interactions (Additional file 3: Script 4). This script determines the shortest path starting from each transcription factor within the general network to each of the genes of interest, following the direction of the regulatory interactions. Then, it outputs the distance in edges for each shortest path. If a transcription factor does not have a directional path towards a gene of interest, that distance is omitted.

### Real time PCR

Purified RNA (500 ng) was used for retrotranscription with the iScript RT Supermix (Biorad, CA). The resulting cDNA was amplified in duplicate by rtPCR with a StepOnePlus thermocycler (Applied Biosystems, CA) using the PowerUp SYBR Green master mix (Thermo, MA) and 100 nM of each primer. The primers, annealing temperatures and amplification efficiencies are listed in Additional file 1: Table S6. The amplification conditions were as follows: 5 min at 95 °C, 40 cycles of 15 s at 95 °C, 15 s of annealing and 30 s at 72 °C. After every reaction, a melting curve was performed to ensure the amplification of a single product. The efficiency of the amplification with each pair of primers was determined by serial dilution of a mixture of the cDNAs. Then, the relative expression was calculated for each sample using the equation by Pfaffl [44] and the TATA-box binding protein (*Tbp*) as reference gene.

### Western blotting

Individual embryos were solubilized in T-PER solution (Thermo, MA) containing cOmplete Protease Inhibitor Cocktail (Roche, Switzerland) on ice by gentle pipetting. After centrifugation at 12000 x g for 10 min at 4 °C, the supernatant was collected and the protein content was estimated using the Protein Assay Kit (Biorad, CA). Since total protein levels obtained from individual embryos were small, we loaded 16 μl of each sample in a 10% polyacrylamide gel for electrophoretic separation at 100 V for around 2 h. Then, proteins were transferred to a nitrocellulose membrane for 1 h at a constant current of 300 mA on ice. Membranes were blotted with antibodies raised against AR (rabbit polyclonal IgG 1:500; Santa Cruz Biotechnologies, TX) and Tubulin (TUBB; rabbit polyclonal IgG 160 ng/ml; Abcam, England), this latter as protein loading control. Antibody binding was detected with a second antibody raised in goat against rabbit IgG bound to peroxidase (1.8 μg/ml; Sigma, MO), revealed by chemiluminescence and documented using a G:Box Chemi XRQ system (Syngene, England). Band intensity was measured with the ImageJ 1.45 software. The intensity of each band was expressed as percentage of the average intensity of WT samples in each gel.

### Data presentation, reduction of bias, and statistics

Data generated by rtPCR is exponential in nature and are presented as the geometric mean + error (uncertainty in calculating the relative expression). Arithmetic data are shown as scatter plots with a horizontal line representing the mean. Expression levels based on sequencing data are shown as heat maps. Mean read counts in each group were log transformed and centered on the grand mean before plotting the heat maps. For enrichment analyses, enrichment fold and *p*-values are plotted side by side for each Gene Ontology term. For correlations, Pearson correlation coefficients are presented in co-variance matrices.

The assignment of pregnant dams to each treatment group was pseudo-randomized. Each day, the first female with a vaginal plug was assigned to the control group, the second one to one of the treatment groups, and so on. If only one female had a plug 1 day, the next day the order was reversed. To reduce bias in sample analysis, embryos were processed in random order and blinding was as follows. By design, phenotypic assessment of embryos was blinded to genotype, but not to maternal treatment. Genotyping was performed blinded to the phenotype. RNA-Seq, sequence analysis, and rtPCR were done blinded to both genotype and phenotype. Western blotting was done without blinding. To reduce bias in gene regulatory network analyses, we defined all the filters a priori and applied them systematically.

The statistical significance of the difference in expression levels determined by mRNA sequencing was evaluated with Cufflinks 2.2.1. The PANTHER implementation of Gene Set Enrichment Analysis tests for statistical significance by calculating a p-value based on a hypergeometric distribution with a Benjamini-Hochberg correction. To assess the significance of the differences between geometric means of relative expression obtained by rtPCR, we used the Relative Expression Software Tool, which implements a Pair-Wise Fixed Reallocation Randomisation Test [45]. To test the significance of the difference between arithmetic means we used ANOVA with Tukey’s post-test. When categorical variables were compared, we used the Fisher’s exact test for dichotomous outcomes, or χ^2^ test for more than 2 possible outcomes. To determine the significance of correlations, confidence intervals for Pearson correlation coefficients were calculated by assuming data followed a Gaussian distribution. Where applicable, tests were two-tailed and results were considered significant at *p* < 0.05.

## Additional files

Additional file 1: **Table S1**. Yield and quality of mRNA sequencing. **Table S2**. Top 10 biological processes enriched in the set of downregulated genes in KO-NTD embryos compared to WT and KO-N embryos. **Table S3**. Top 10 biological processes enriched in the set of upregulated genes in KO-NTD embryos compared to WT and KO-N embryos. **Table S4**. Topological features of the mouse background network and the networks constructed in this analysis. **Table S5**. Shortest paths from transcription factors to the genes of interest within the general network. **Table S6**. Sequence and reaction conditions of the primers used in real time PCR assays. **Figure S1**. Frequency of numbers of shared transcription factors (TF) in all the possible subnetworks from the KO-N vs. KO-NTD regulatory network. **Figure S2**. Expression levels of the Ar gene in the RNA-Seq data. **Figure S3**. Expression domains of selected genes. **Figure S4**. Regulatory circuit in humans. (DOCX 572 kb)

Additional file 2: Differentially regulated genes in WT, KO-N and KO-NTD embryos. (XLSX 166 kb)

Additional file 3: Perl scripts 1–4. (ZIP 9 kb)

Additional file 4: Cytoscape session containing the General network. (CYS 134 kb)

Additional file 5: Cytoscape sessions for each of the subnetworks. (ZIP 258 kb)

## Abbreviations

Alx1: ALX homeobox 1
Alx3: ALX homeobox 3
AMN: amnionless
ApoA-I: Apolipoprotein A-I
ApoA-IV: Apolipoprotein A-IV
Apob: Apolipoprotein B
Apoc1: Apolipoprotein C-I
ApoE: Apolipoprotein E
ApoM: Apolipoprotein M
Ar: Androgen receptor
Cebpb: CCAAT/enhancer binding protein beta
CUBN: Cubilina
HDL: High density lipoprotein
KO: Knock-out
LRP-2: Lipoprotein receptor-related protein 2
LRP-8: Llipoprotein receptor-related protein 8
Marcks: Myristoylated alanine-rich C kinase substrate
MTTP: Microsomal triglyceride transfer protein
Neurog2: Neurogenin 2
Nfkb1: Nuclear factor of kappa light polypeptide gene enhancer in B cells 1, p105
NTD: Neural tube defects
Pax3: Paired box 3
Ppara: Peroxisome proliferator activator receptor alpha
RBP: Retinol binding protein
RFC1: Reduced folate carrier 1
rtPCR: Real-time polymerase chain reaction
SOAT1: Sterol O-acyltransferase 1
SOAT2: Sterol O-acyltransferase 2
SR-B1: Scavenger receptor class B type 1
Tbp: TATA-box binding protein
WT: Wild-type
