# Supplementary material for: Transcriptional profiling of embryos lacking the lipoprotein receptor SR-B1 reveals a regulatory circuit governing a neurodevelopmental or metabolic decision during neural tube closure
Source: BMC Genomics. 2018 Oct 5;19:731. doi: 10.1186/s12864-018-5110-2 (PMC6173885; doi:10.1186/s12864-018-5110-2)
Supplement: Supplementary file 1 — Table S1. Yield and quality of mRNA sequencing. Table S2. Top 10 biological processes enriched in the set of downregulated genes in KO-NTD embryos compared to WT and KO-N embryos. Table S3. Top 10 biological processes enriched in the set of upregulated genes in KO-NTD embryos compared to WT and KO-N embryos. Table S4. Topological features of the mouse background network and the networks constructed in this analysis. Table S5. Shortest paths from transcription factors to the genes of interest within the general network. Table S6. Sequence and reaction conditions of the primers used in real time PCR assays. Figure S1. Frequency of numbers of shared transcription factors (TF) in all the possible subnetworks from the KO-N vs. KO-NTD regulatory network. Figure S2. Expression levels of the Ar gene in the RNA-Seq data. Figure S3. Expression domains of selected genes. Figure S4. Regulatory circuit in humans. (DOCX 572 kb) [file 12864_2018_5110_MOESM1_ESM.docx]

**Table S1. Yield and quality of mRNA sequencing.**

| **Sample** | **Group** | **Read number** | **Yield (Gbases)** | **Mean quality score** |
| --- | --- | --- | --- | --- |
| 1 | WT | 44862107 | 2.243 | 39.69 |
| 2 | KO-N | 46418607 | 2.321 | 39.70 |
| 3 | KO-NTD | 41455587 | 2.073 | 39.69 |
| 4 | WT | 43471850 | 2.174 | 39.69 |
| 5 | KO-N | 40884082 | 2.044 | 39.70 |
| 6 | KO-NTD | 42123725 | 2.106 | 39.69 |
| 7 | WT | 45489387 | 2.274 | 39.67 |
| 8 | KO-N | 43663253 | 2.183 | 39.69 |
| 9 | KO-NTD | 40099540 | 2.005 | 39.69 |

**Table S2. Top 10 biological processes enriched in the set of downregulated genes in KO-NTD embryos compared to WT and KO-N embryos.**

| **Biological process** | **Observed genes** | **Expected genes** | **Enrichment** | **P-value** |
| --- | --- | --- | --- | --- |
| Cerebral cortex regionalization (GO:0021796) | 4 | 0.1 | 39.19 | 3.5E-02 |
| Metanephric nephron tubule development (GO:0072234) | 5 | 0.15 | 33.91 | 4.2E-03 |
| Glial cell fate commitment (GO:0021781) | 5 | 0.16 | 31.49 | 6.1E-03 |
| Dorsal spinal cord development (GO:0021516) | 5 | 0.23 | 22.04 | 3.4E-02 |
| Neuron fate specification (GO:0048665) | 8 | 0.41 | 19.6 | 1.0E-04 |
| Cerebral cortex radially oriented cell migration (GO:0021799) | 7 | 0.41 | 17.15 | 2.1E-03 |
| Cell proliferation in forebrain (GO:0021846) | 6 | 0.39 | 15.56 | 2.7E-02 |
| Cell differentiation in spinal cord (GO:0021515) | 9 | 0.6 | 14.97 | 1.2E-04 |
| Forelimb morphogenesis (GO:0035136) | 7 | 0.5 | 14.03 | 7.9E-03 |
| Ventral spinal cord development (GO:0021517) | 7 | 0.54 | 12.86 | 1.4E-02 |

**Table S3. Top 10 biological processes enriched in the set of upregulated genes in KO-NTD embryos compared to WT and KO-N embryos.**

| **Biological process** | **Observed genes** | **Expected genes** | **Enrichment** | **P-value** |
| --- | --- | --- | --- | --- |
| High-density lipoprotein particle assembly (GO:0034380) | 4 | 0.07 | 59.73 | 6.6E-03 |
| Phospholipid efflux (GO:0033700) | 4 | 0.09 | 43.44 | 2.3E-02 |
| Reverse cholesterol transport (GO:0043691) | 4 | 0.11 | 36.76 | 4.4E-02 |
| Branching involved in labyrinthine layer morphogenesis (GO:0060670) | 4 | 0.11 | 36.76 | 4.4E-02 |
| Cholesterol efflux (GO:0033344) | 7 | 0.2 | 34.84 | 1.7E-05 |
| Plasma lipoprotein particle remodeling (GO:0034369) | 5 | 0.16 | 31.44 | 6.0E-03 |
| Positive regulation of fatty acid biosynthetic process (GO:0045723) | 5 | 0.18 | 28.44 | 9.8E-03 |
| Negative regulation of endothelial cell apoptotic process (GO:2000352) | 6 | 0.23 | 25.6 | 1.5E-03 |
| Vitamin transport (GO:0051180) | 6 | 0.23 | 25.6 | 1.5E-03 |
| Labyrinthine layer blood vessel development (GO:0060716) | 5 | 0.22 | 22.97 | 2.8E-02 |

**Table S4. Topological features of the mouse background network and the networks constructed in this analysis.**

| **Property** | **RegNetwork** | **KO-N vs. KO-NTD** | **Apoa1** | **Apob** | **Apoe** | **Pax3** |
| --- | --- | --- | --- | --- | --- | --- |
| Nodes | 20738 | 641 | 34 | 18 | 9 | 24 |
| Clustering coefficient | 0.101 | 0.048 | 0.182 | 0.331 | 0.193 | 0.132 |
| Connected components | 1 | 5 | 1 | 1 | 1 | 1 |
| Network diameter | 8 | 9 | 6 | 3 | 3 | 5 |
| Shortest paths | 36743196 | 18038 | 508 | 53 | 30 | 177 |
| Characteristic path length | 3.229 | 3.488 | 2.469 | 1.377 | 1.733 | 2.215 |
| Average number of neighbors | 30.55 | 5.86 | 5 | 4 | 2.67 | 4.42 |

**Table S5. Shortest paths from transcription factors to the genes of interest within the general network.**

| **TF** | **Distance to** | | | | **Total distance** |
| --- | --- | --- | --- | --- | --- |
|  | **Apoa1** | **Apob** | **Apoe** | **Pax3** |  |
| *Ar* | 2 | 1 | 3 | 2 | 8 |
| *Ppara* | 1 | 1 | 3 | 3 | 8 |
| *Nfkb1* | 1 | 3 | 3 | 2 | 9 |
| *Rela* | 2 | 2 | 3 | 2 | 9 |
| *Creb1* | 2 | 2 | 3 | 3 | 10 |
| *Rarb* | 2 | 3 | 4 | 2 | 11 |
| *Stat3* | 3 | 3 | 3 | 2 | 11 |
| *Mapk10* | 2 | 3 | 4 | 3 | 12 |
| *Mapk8* | 2 | 3 | 4 | 3 | 12 |
| *Mapk9* | 2 | 3 | 4 | 3 | 12 |

**Table S6. Sequence and reaction conditions of the primers used in real time PCR assays.**

| **Gene** |  | **Primer** | **Annealing T°** | **Efficiency** |
| --- | --- | --- | --- | --- |
| *Tbp* | F | CAGATGTGCGTCAGGCGTTC | 57°C | 1.93 |
|  | R | AGAAACCTAGCCAAACCGCC |  |  |
| *Alx1* | F | AAAGAGAACGATACGGCCAA | 54°C | 2.2 |
|  | R | GTTCTGGATCTGTGGGTAGC |  |  |
| *Alx3* | F | CATCCTCAGCTGCAGAACTC | 54°C | 2.08 |
|  | R | GTATGGGGACATGCATGGAG |  |  |
| *Neurog 2* | F | TTAACTGGAGTGCCTTGGAGT | 54°C | 1.96 |
|  | R | GTTGTCGTTCTCGTGCGT |  |  |
| *Pax3* | F | GCAATGGCCTTTCACCTCAG | 57°C | 2 |
|  | R | AGGGGAGAGAGCATAGTCGG |  |  |
| *Apoa1* | F | AGAAGAGCTGGACACCCAGA | 54°C | 1.82 |
|  | R | CACCACAGCTTTCATCCTGA |  |  |
| *Apob* | F | CAAGCTGGCATAAGAACCAA | 53°C | 1.83 |
|  | R | CCATCCTGAGTTGGACAAAAC |  |  |
| *Apoe* | F | TGTTTCGGAAGGAGCTGACT | 54°C | 1.82 |
|  | R | AGCAATGTGACCAACAGCAC |  |  |
| *Amn* | F | GATTTTCGACGCGATTGTCT | 54°C | 1.99 |
|  | R | AAGAGTGGGTTAACGAAGCA |  |  |
| *Cubn* | F | CATTTGGTGTTCTGAAGGCA | 54°C | 2.02 |
|  | R | AGGTCTCCACTAAATGGTGC |  |  |
| *Lrp2* | F | TAGGGCTTTGGGTCTCTCTA | 54°C | 2.2 |
|  | R | AAACGGACCCACAAATGAAG |  |  |
| *Ar* | F | TTCCAATCCTGGTTGAGTGT | 54°C | 1.8 |
|  | R | ACCCAGTCAATCCTTTCTGT |  |  |


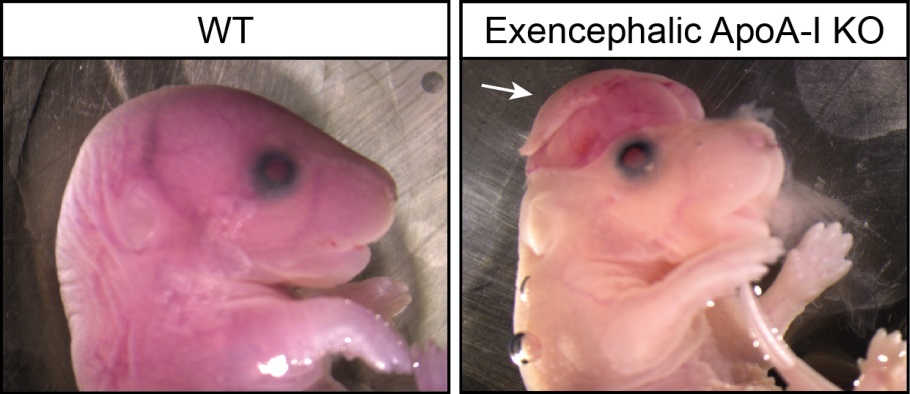


**Figure S1. Exencephaly in fetuses lacking ApoA-I.** Mouse fetuses were recovered at E18.5 from wild-type (WT) or ApoA-I KO intercrosses, and the presence of NTD was evaluated. An exposed brain (exencephaly, white arrow) was observed in 2 of the 51 analyzed ApoA-I KO fetuses, but is not observed in WT fetuses.


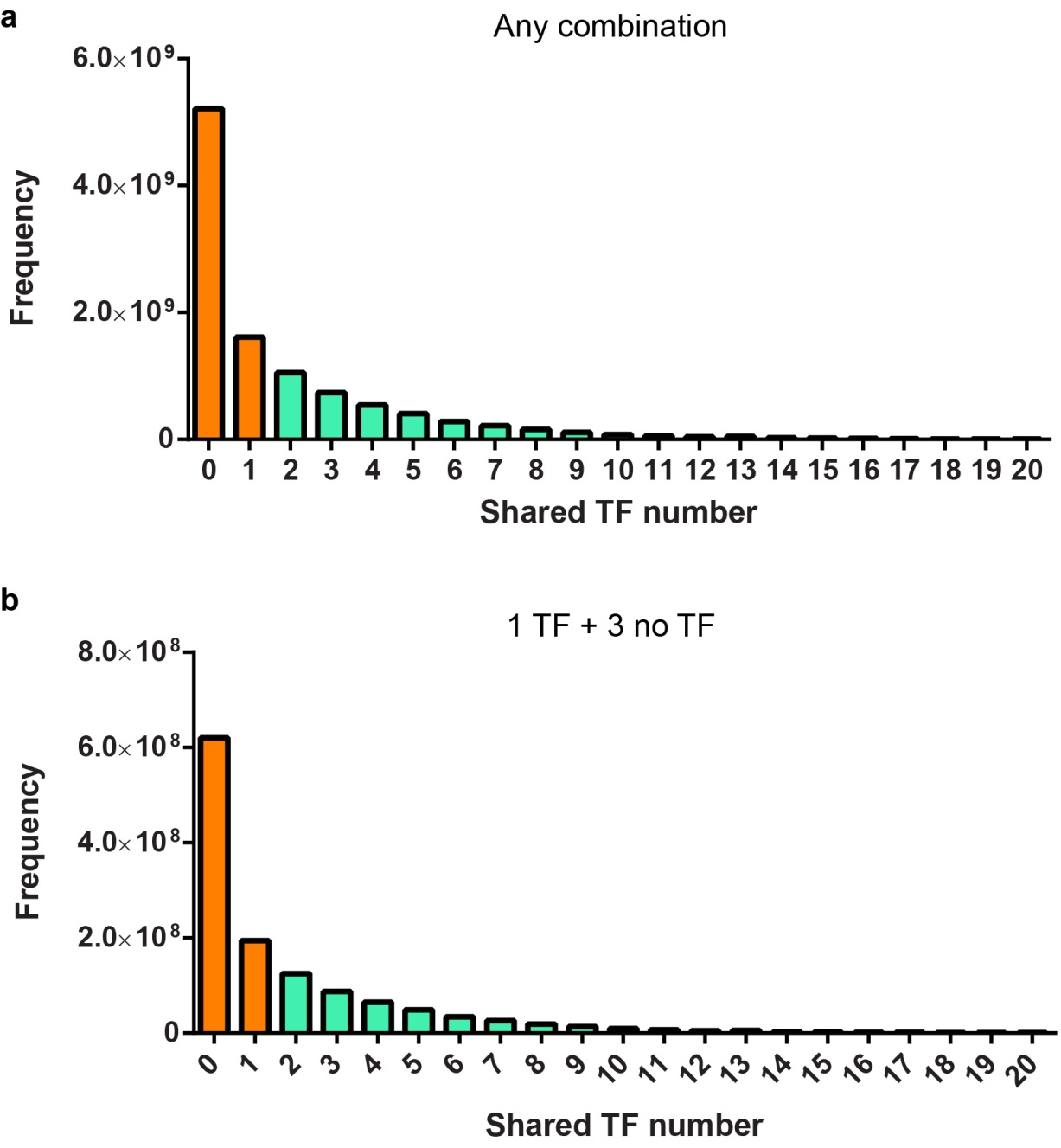


**Figure S2. Frequency of numbers of shared transcription factors (TF) in all the possible subnetworks from the KO-N vs. KO-NTD regulatory network.** We constructed all the possible quadruplets of subnetworks following the same protocol as described for the genes of interest, and determined the TF shared in all the subnetworks. We used any combination of seed nodes (**a**) or only 1 TF and 3 genes not classified as TF (**b**). In both cases, 73% of the quadruplets shared 0 or 1 TF (orange bars), while only 27% of the quadruplets showed 2 or more shared TF (teal bars), the number of shared TF in the subnetworks using the genes of interest as seed nodes.


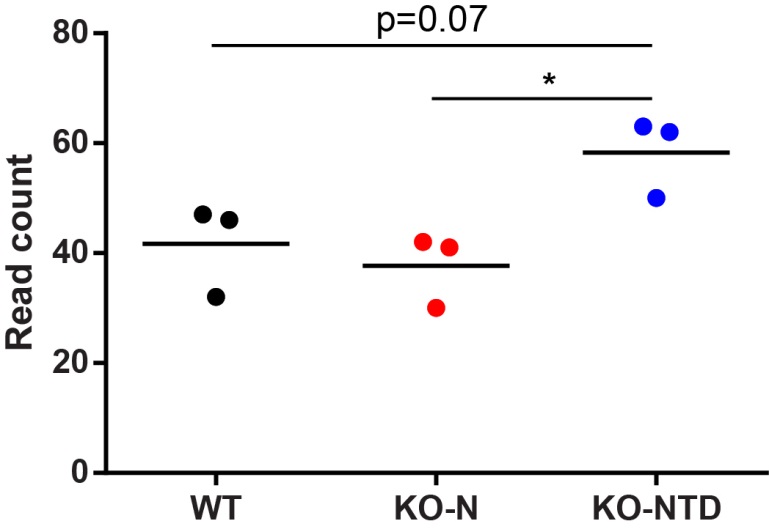


**Figure S3. Expression levels of the Ar gene in the RNA-Seq data.** Number of reads mapping to the Ar gene in each group of embryos is shown. Differences were not statistically significant at the transcriptome level. Statistical analysis taking only the Ar gene into account is shown in the figure. *p<0.05; ANOVA with Tukey post-hoc test.


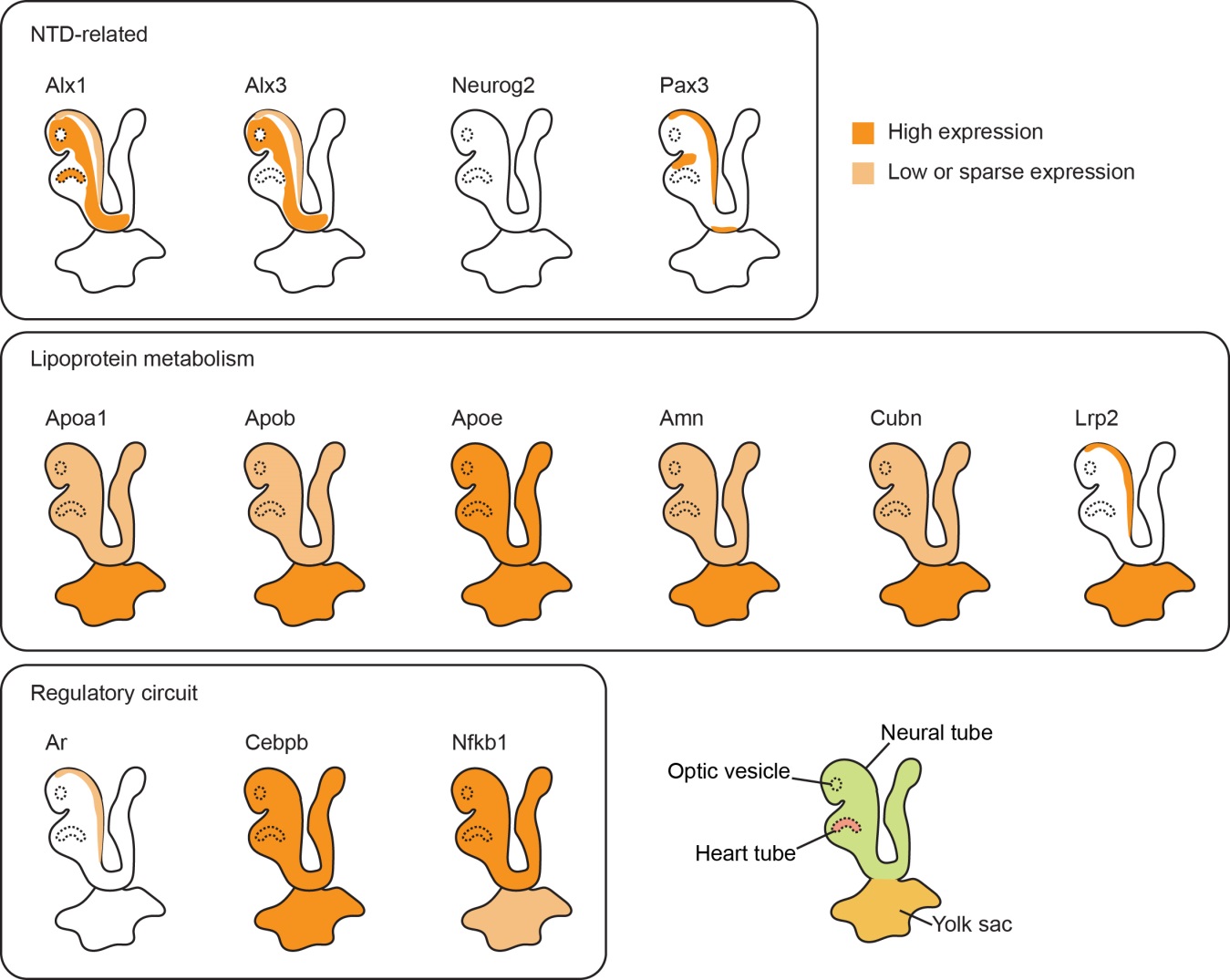


**Figure S4. Expression domains of selected genes.** The approximate expression domains of the genes of each category in E8.25 embryos was extracted from the database created by Ibarra-Soria et al., using their online tool at http://marionilab.cruk.cam.ac.uk/organogenesis/ (accessed on 02-05-2018).

**
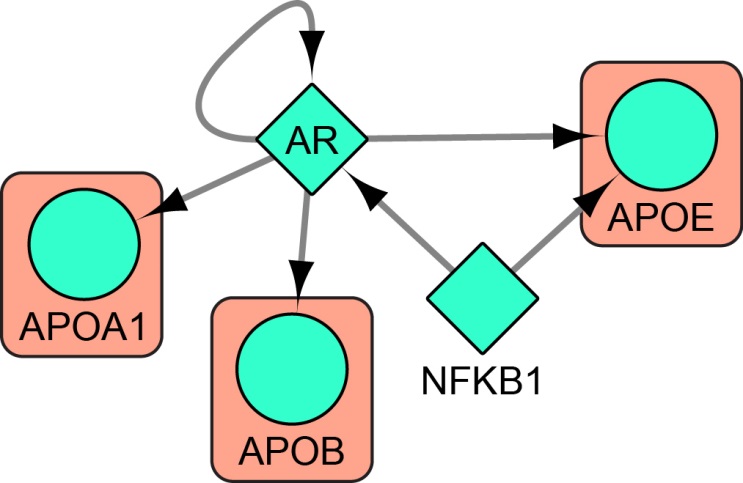
**

**Figure S5. Regulatory circuit in humans.** Nodes of the components of the regulatory circuit (teal diamonds) and the genes of interest (teal circles in red boxes) were extracted from human background network, based on experimental data in RegNetwork.
